# Human Cerebral Organoids and Fetal Brain Tissue Share Proteomic Similarities

**DOI:** 10.3389/fcell.2019.00303

**Published:** 2019-11-28

**Authors:** Juliana Minardi Nascimento, Verônica M. Saia-Cereda, Rafaela C. Sartore, Rodrigo Madeiro da Costa, Clarissa S. Schitine, Hercules Rezende Freitas, Michael Murgu, Ricardo A. de Melo Reis, Stevens K. Rehen, Daniel Martins-de-Souza

**Affiliations:** ^1^Laboratory of Neuroproteomics, Department of Biochemistry and Tissue Biology, Institute of Biology, University of Campinas (UNICAMP), Campinas, Brazil; ^2^D’Or Institute for Research and Education (IDOR), Rio de Janeiro, Brazil; ^3^National Institute of Traumatology and Orthopedics, Rio de Janeiro, Brazil; ^4^Institute of Biomedical Sciences, Federal University of Rio de Janeiro (UFRJ), Rio de Janeiro, Brazil; ^5^Institute of Biophysics, Federal University of Rio de Janeiro (UFRJ), Rio de Janeiro, Brazil; ^6^School of Health Sciences, IBMR – University Center, Rio de Janeiro, Brazil; ^7^Waters Corporation, Barueri, Brazil; ^8^Instituto Nacional de Biomarcadores em Neuropsiquiatria (INBION), Conselho Nacional de Desenvolvimento Científico e Tecnológico, São Paulo, Brazil; ^9^Experimental Medicine Research Cluster (EMRC), University of Campinas, Campinas, Brazil

**Keywords:** brain organoids, neural cells, proteomics, oligodendrocyte progenitors, stem cells

## Abstract

The limited access to functional human brain tissue has led to the development of stem cell-based alternative models. The differentiation of human pluripotent stem cells into cerebral organoids with self-organized architecture has created novel opportunities to study the early stages of the human cerebral formation. Here we applied state-of-the-art label-free shotgun proteomics to compare the proteome of stem cell-derived cerebral organoids to the human fetal brain. We identified 3,073 proteins associated with different developmental stages, from neural progenitors to neurons, astrocytes, or oligodendrocytes. The major protein groups are associated with neurogenesis, axon guidance, synaptogenesis, and cortical brain development. Glial cell proteins related to cell growth and maintenance, energy metabolism, cell communication, and signaling were also described. Our data support the variety of cells and neural network functional pathways observed within cell-derived cerebral organoids, confirming their usefulness as an alternative model. The characterization of brain organoid proteome is key to explore, in a dish, atypical and disrupted processes during brain development or neurodevelopmental, neurodegenerative, and neuropsychiatric diseases.

## Introduction

Understanding the molecular basis of human diseases is currently one of the main challenges faced in contemporary medicine. Particularly regarding the human central nervous system (CNS), the challenge lies in both its complexity and the reduced accessibility to living tissue. Research with postmortem brains and animal models along with a broader spectrum of tools, such as brain imaging and studies utilizing pluripotent stem cells (PSC) – have all advanced understanding in the field. Differentiating PSC, both human embryonic stem cells (hESC) (Thomson, 1998), and induced pluripotent stem cells (iPSC) (Takahashi et al., 2007), into functional neural cells grown in 2D cultures (Zhang et al., 2001; Chambers et al., 2009), and 3D brain organoid cultures (Eiraku and Sasai, 2012; Lancaster et al., 2013) has become a functional alternative to investigate CNS disorders.

Hence, PSC-derived cerebral organoids have recently become a focus in the complex disorders quest. Upon minimal instructions *in vitro*, cells can self-organize into 3D structures where intrinsic molecular programs are activated to generate diverse neuronal and non-neuronal cell types from CNS specific regions, recapitulating some of the early features of the human brain development (Lancaster et al., 2013; Sasai, 2013; Lancaster and Knoblich, 2014; Quadrato et al., 2017; Sartore et al., 2017). Some recent studies have shown potential for modeling several human disorders using cerebral organoids, such as microcephaly (Lancaster et al., 2013), lissencephaly (Bershteyn et al., 2017), Zika virus infection microcephaly (Cugola et al., 2016; Garcez et al., 2016; Qian et al., 2016), Alzheimer’s disease (Raja et al., 2016), and autism spectrum disorders (Mariani et al., 2015). Those modeling applications may lead to new insight into drug discovery and interactions, cell therapy, and basic research (Brennand et al., 2014; Quadrato and Arlotta, 2017).

Advances in 3D cell–cell interaction using cellular and molecular tools have shown a diverse and more advanced cell maturation profile within brain organoids (Paşca et al., 2015; Quadrato et al., 2017; Sloan et al., 2017; Madhavan et al., 2018; Ormel et al., 2018). Notable similarities in cell composition and gene expression profiles between *in vitro* cortical development of human brain organoids and human fetal neocortex were revealed by single-cell RNA sequencing and whole-organoid transcriptomics (Camp et al., 2015; Bershteyn et al., 2017; Xiang et al., 2017). However, oftentimes mRNA levels poorly correlate to cell expression of selected markers, due to divergences in translation (Carlyle et al., 2017). Regarding proteins, only a small number have been identified by immunocytochemistry in brain organoids, most of which are considered regional patterning cell markers (Renner et al., 2017).

However, fundamental organization of the developing brain is orchestrated by thousands of molecules simultaneously. And understanding those broad and complex molecular processes are key to unravel novel targets in disease modeling. As such, mass spectrometry-based proteomics can offer a complementary outlook to mRNA, as a great molecular tool to uncover deeper and more comprehensive large-scale, protein-level data. The possibility of detecting thousands of proteins within a sample at a given moment can reveal functional profiles associated with genes and interaction with their environment.

Here we describe in what ways stem cell-derived cerebral organoids is similar to brain tissue in proteomic terms, defending its use as a robust model to study psychiatric disorders. We present findings on large-scale proteome profiling of human cerebral organoids, using systems level analysis showing initial development of varied cell types leading to a complex neural network. This includes proteins of a wide-range of cellular functions, indicative of active pathways in organoids, including neuritogenesis, dendritic branching and synapse formation, initial gliogenesis, and oligodendrogenesis. The protein data provide a deeper knowledge of the microenvironmental niches governing a network of functional molecules in organoid neocortical development. Molecular information from protein levels, signaling, and pathways of interest can be used to better assess neurodevelopmental abnormalities in further investigations. Complex brain disorders such as schizophrenia, bipolar disorder, autism, and Alzheimer’s disease benefit from studies on unbiased cellular and molecular interactions, where models and hypotheses can be further tested to uncover disrupted processes.

## Materials and Methods

### Human Pluripotent Stem Cells and Cerebral Organoids Differentiation

Human embryonic stem cells [hESC; cell line BR1 obtained from the Laboratory for Embryonic Stem Cell Research (LaNCE), University of São Paulo (Fraga et al., 2011)] were cultured in mTeSR1 media (Stemcell Technologies, Vancouver, BC, Canada) on Matrigel (BD Biosciences)-coated cell culture plates. The hESC colonies were manually passaged upon 70% confluence and maintained at 37°C in humidified air with 5% CO_2_. The differentiation of PSC into cerebral organoids was performed as previously described (Sartore et al., 2017). Concisely, cells were inoculated in a spinner flask containing mTeSR1 media supplemented with 10 μM Y-27632 (Rho-associated protein kinases inhibitor, iRock) (Merck) under constant rotation (40 rpm). After 24 h, cell culture medium was replaced to initiate embryoid body formation. By day 7, neural induction media [DMEM/F12 1:1 supplemented with N2 (1x) supplement, 2 mM glutamax, 1% MEM-NEAA (Thermo Scientific), and heparin (1 μg/mL, Sigma)] was added. On day 11, cellular aggregates were covered in Matrigel and cultured in differentiation media [DMEM/F12:Neurobasal (1:1), supplemented with N2 (0.5x) and B27 minus vitamin A (1x) supplements, 2 mM glutamax, 0.5% MEM-NEAA 0.2 μM 2-mercaptoethanol (Thermo Scientific), and 2.5 μg/mL insulin (Sigma)] for 4 days. After this period, the medium was replaced with the same formulation, except with 1x B27 containing vitamin A (Thermo Scientific). This final differentiation medium was replaced every week during the complete differentiation process (45 days).

### Sample Preparation and Digestion

Two separate hESC (BR1) cultures were harvested at 70–80% confluence. At 45 days in culture, five hESC-derived cerebral organoids were pooled to provide population variability within each experiment. Three different experimental spinner flasks, differentiated by distinct differentiation processes (batches), were analyzed. Cell lysates of either hESC or 45-day organoids were homogenized in extraction buffer containing 7 M urea, 2 M thiourea, 4% CHAPS, 70 mM DTT and EDTA-free protease inhibitor cocktail (Roche). Sample lysates were centrifuged at 10,000 × *g* for 10 min at 4°C. The supernatant was collected and quantified with a Qubit^®^ 3.0 Fluorometer (Thermo Fisher Scientific). Each sample (50 μg) was subjected to an SDS-PAGE gel electrophoresis and *in gel* reduction, alkylation, and overnight digestion in a 1:50 (trypsin:total protein) solution, at 37°C. Peptides obtained from this process were dried by SpeedVac (Thermo Fisher Scientific) and stored at −80°C prior to quantitative and qualitative analyses by shotgun mass spectrometry.

### Liquid Chromatography-Mass Spectrometry

Proteomic analyses were performed on a state-of-the-art 2D-LC-MS/MS system. Peptides were injected into a two-dimensional, liquid chromatographer [Acquity UPLC M-Class System (Waters Corporation, Milford, MA, United States)] coupled to a Synapt G2-S mass spectrometer (Waters Corporation, Milford, MA, United States). In first-dimension reverse-phase chromatography, peptides (5 μg) were loaded onto a M-Class BEH C18 Column (130 Å, 5 μm, 300 μm × 50 mm, Waters Corporation, Milford, MA, United States). Fractionation was performed using ascending concentration steps of acetonitrile (11, 14, 17, 20, and 50% acetonitrile). Peptide loads were directed to second-dimension separation, on a nanoACQUITY UPLC HSS T3 Column (100 Å, 1.8 μm, 75 μm × 150 mm, Waters Corporation, Milford, MA, United States), with an acetonitrile gradient of 7–40% (v/v) over 54 min at a flow rate of 0.4 μL/min directly into a Synapt G2-S. The mass spectrometer acquired data in data-independent mode (DIA) with ion mobility separation (high-definition data-independent mass spectrometry; HDMS^E^), to distinguish ions with the same intact mass, significantly enhancing the proteome coverage (Distler et al., 2014). Injection was performed using nano-electrospray ionization in positive ion mode, nanoESI (+), with a NanoLock Spray (Waters, Manchester, United Kingdom) ionization source. The lock mass channel was sampled every 30 s. Calibration was performed with an MS/MS spectrum of [Glu1]-Fibrinopeptide B human (Glu-Fib) solution from the reference NanoLock Spray source. Each of the three independent biological samples was run in technical triplicates.

### Database Search and Quantification

Raw data was processed with Progenesis^®^ QI for Proteomics, version 3.0 (Waters). Data processing for protein identification and quantification was performed using dedicated algorithms, searching against the Uniprot human proteomic database (version 2017/10), with the default parameters for ion accounting and quantitation (Li et al., 2009). The databases used were reversed “on the fly” during queries and appended to the original database to assess the false-positive identification rate, and false discovery rate (FDR) was set to less than 1%. Other variable parameters set for peptide identification were: up to two missed cleavages for trypsin digestion; variable modifications by oxidation (M) and fixed modifications by carbamidomethyl (C). Identifications not satisfying these criteria were rejected. The quantitative analysis was carried out on the log2-values of the measured intensities. Raw mass spectrometry (MS) files used in this experiment were uploaded to the PRIDE proteomics data repository with the accession number PXD011605.

### *In silico* Analysis

Gene ontology was analyzed using DAVID^1^ (Huang et al., 2009a, b) and Panther^2^ (Mi et al., 2016) databases. The significant biological functions are based on Fisher’s exact test. Gene Analytics^3^ and LifeMap^4^ were used to search for specific proteins within cell types. Reactome Pathways Knowledgebase^5^ (Fabregat et al., 2017) and the KEGG knowledge base^6^ (Kanehisa et al., 2016) were used to identify overrepresented pathways. Protein interaction networks were identified using the STRING database^7^ (Szklarczyk et al., 2018). Ingenuity^®^ Pathway Analysis (Qiagen Bioinformatics, Redwood, CA, United States) performed a core analysis on diseases and biofunctions. The BrainSpan Atlas of the Developing Human Brain was also consulted for developmental information^8^. The BrainSpan human developmental transcriptome dataset (RNA-Seq Genecode v10 summarized to genes) Log2 values averaged to genes was compared to the proteomics data of this study. As well, the Log2 transformed values of single-cell transcriptomics data of human cerebral organoids accession GSE75140 and GSE82022 (Camp et al., 2015; Luo et al., 2016), using Spearman correlation. The protein level Log2 transformed values of formalin-fixed, paraffin-embedded human brain data at 16–20 gestational weeks (GW) from the study of Djuric et al. (2017) was compared with data of human cerebral organoids of our study.

### Oligodendrocyte Progenitor Cell in Cerebral Organoids

Cerebral organoids that had been differentiated for 45 days were used for oligodendrocyte progenitor cell (OPC) isolation and initial differentiation. Approximately five cerebral organoids were mechanically dissociated and plated in 100-mm culture dishes (∼5 × 10^6^ cells/dish), previously coated with polyornithine (1.5 μg/mL, Sigma) and laminin (5 μg/mL, Sigma). They were then maintained for 4 days in DMEM/F12 (Invitrogen) supplemented with 1x N2, 0.5x B27, 20 ng/mL of FGF2, and 20 ng/mL of EGF (Thermo Fisher Scientific). After this time, the medium was changed to include 10 ng/mL of platelet-derived growth factor-AA (PDGFA, Thermo Fisher Scientific) and incubated for another week. Upon confluence, cells were passaged, and the medium was replaced with DMEM/F12 containing 0.5x B27, 10 ng/mL PDGFA, 30 ng/mL T3, 10 ng/mL insulin-like growth factor 1 (IGF-1), and 200 μM ascorbic acid (Sigma-Aldrich). This medium was changed every 5 days for another 8 weeks, when OPCs showed initial ramified morphology and were then analyzed by immunolabeling.

### Single-Cell Calcium Imaging

Variations of free intracellular calcium ([Ca^2+^]i) levels were evaluated in single cells obtained from dissociated cerebral organoids in culture, following application of KCl or ATP using a method adapted from the protocol of retina cells (Freitas et al., 2016) or cortical astrocytes (Faria et al., 2016). Dissociated organoids (30 and 45-days) were plated onto a 15-mm coverslip (Marienbad, Germany) and maintained for 10 days *in vitro* to allow adhesion and network stabilization. Prior assessment, cells in culture were loaded for 40 min with 5 μM Fura-2/AM (Molecular Probes), 0.1% fatty acid-free bovine serum albumin (BSA), and 0.02% pluronic F-127 (Molecular Probes) in Krebs solution (132 mM NaCl, 4 mM KCl, 1.4 mM MgCl_2_, 2.5 mM CaCl_2_, 6 mM glucose, 10 mM HEPES, pH 7.4), in an incubator with 5% CO_2_ at 37°C. After a 10-min post-loading period at room temperature in Krebs solution, to obtain a complete hydrolysis of the probe, coverslips with the cells were mounted on a chamber in a PH3 platform (Warner Instruments, Hamden, CT, United States) for rapid perfusion on the stage of an inverted fluorescence microscope (Axiovert 200; Carl Zeiss). Cells were continuously perfused with Krebs solution (273 mOsm) and stimulated with different solutions (50 mM KCl or 100 μM ATP). The solutions were prepared immediately before the assays. Preparing 50 mM KCl (Sigma) 55.9 mg were diluted in 15 mL Krebs, 372 mOsm; while for ATP (Sigma) 92 mg were diluted in 1 mL (preparing a 167 mM stock solution) we then diluted to a 10 mL ATP, to 100 μM, final pH set to 7.4. Solutions were added to the cells by a fast-transition system (approximately 8 s). The variations in [Ca^2+^]i were evaluated by quantifying the ratio of fluorescence emitted at 510 nm following alternate excitation (750 ms) at 340 and 380 nm, using a Lambda DG4 apparatus (Sutter Instrument, Novato, CA, United States) and a 510 nm long-pass filter (Carl Zeiss) before fluorescence acquisition with a 40X objective by Cool SNAP digital camera (Roper Scientific, Trenton, NJ, United States). Acquired values were processed using MetaFluor software (Universal Imaging Corp., West Chester, PA, United States). Values for Fura-2 fluorescence ratio were calculated based on a 15% increase cutoff of the [Ca^2+^]i level induced by the stimulus. Cell cultures after single cell imaging were fixed in 4% paraformaldehyde (PFA) for immunolabeling.

### Immunohistochemistry and Immunocytochemistry

Cerebral organoids were collected from spinner flasks and fixed in 4% PFA, followed by incubation in sucrose solutions over an increasing gradient (10, 20, and 30%) prepared in phosphate buffered saline (PBS). Organoids were then embedded in optimal cutting temperature compound (OCT) and frozen in liquid nitrogen. The organoids were sectioned with a cryostat (Leica) into 20 μm thick sections.

Dissociated cerebral organoids and OPCs were fixed in 4% PFA. After fixed, cells were washed with PBS, permeabilized in 0.3% Triton-X solution, blocked in a 3% bovine serum albumin solution (BSA), and immunolabeled using primary antibodies: anti-Nestin (1:100, MAB5326, Millipore), anti-Neurofilament 200 (1:400, N0142, Sigma-Aldrich), anti-class III β-tubulin (1:100, MAB1637, Millipore), anti-S100β (1:200, ab52642, Abcam). For isolated and differentiated OPCs, primary antibodies were: anti-BLBP (1:200, AB110099, Abcam), anti-CNPase (1:200, #5664, Cell Signaling), anti-PDGFRA (1:1000, #3174, Cell Signaling), anti-olig2 (1:100, MABN50, Millipore), and anti-PAX6 (1:200, sc-11357, KloneLife). Secondary antibodies Alexa Fluor 488 goat anti-mouse (A11001, Invitrogen) and goat anti-rabbit (A11008), and Alexa Fluor 594 goat anti-mouse (A11032; Invitrogen) were used. DAPI (4′, 6- diamidino-2-phenylindole, 1 mg/mL) was used for nucleus staining. After immunostaining, cerebral organoids and OPCs were visualized using a Leica SP5 confocal microscope or a Leica DM5500B fluorescence microscope (Leica, Germany).

### Transmission Electron Microscopy

Organoids were fixed in a solution of 2.5% glutaraldehyde in 0.1 M cacodylate buffer (v/v), post-fixed for 5 min in 1% OsO_4_ solution in cacodylate buffer (w/v) containing 5 mM CaCl_2_ and 0.8% potassium ferricyanide (w/v). Samples were dehydrated in solutions of increasing acetone concentration and embedded in epoxy resin (EMS, PA, United States). Ultra-thin (70 nm) sections were stained with uranyl acetate and lead citrate and observed with a Leo 900 electron microscope at 80 kV (Zeiss, Germany).

## Results

### Proteome Enrichment of PSC-Derived Cerebral Organoids

**FIGURE 1:**
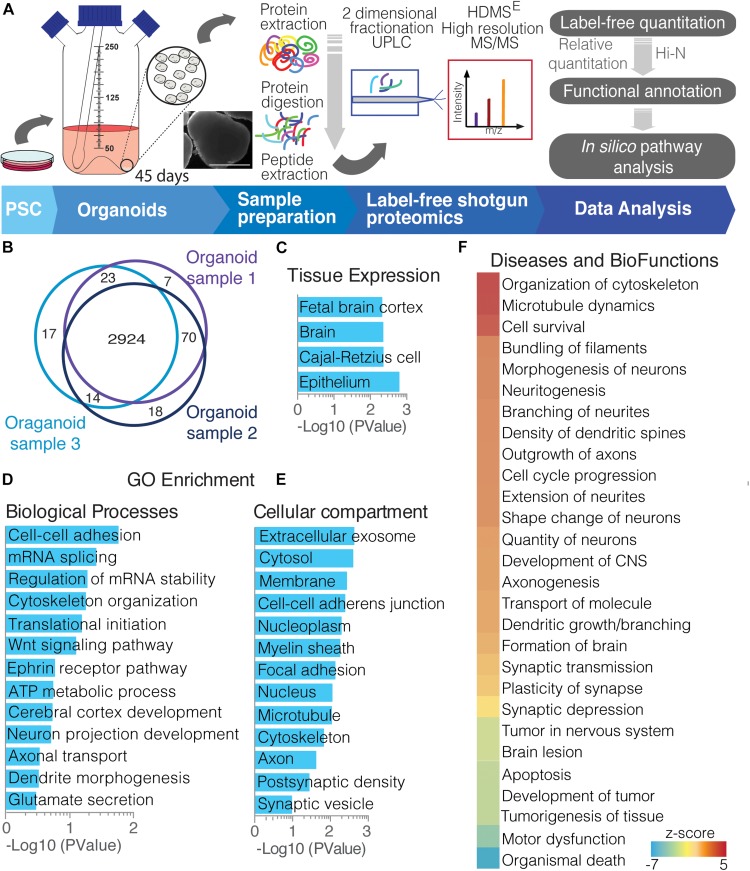
Overview of cerebral organoid protein expression. **(A)** Experimental proteomics workflow, from collecting cerebral organoids and processing to proteomic studies. After sample preparation (extraction and digestion), peptides underwent a 2D-UPLC fractionation and on-line detection using HDMS^E^ high resolution MS/MS acquisition. The acquired peptides were deconvoluted, identified, and quantified using the software Progenesis, whereupon proteins were identified and quantified. This was followed by functional annotation of protein groups and other *in silico* analyses. **(B)** Venn diagram showing commonly found proteins among cerebral organoids replicates (PSC-derived samples). Functional annotation analyses of proteins commonly found in replicates are clustered by **(C)** tissue expression; gene ontology using **(D)** biological function and **(E)** cellular compartment distribution; and **(F)** diseases and biofunctions activity.

To resolve the complex proteome of whole, 45-day cerebral organoids, we used state-of-the-art label-free shotgun proteomics to provide broader coverage of cerebral organoids cultured for 45 days (Figure 1A), in which early neuronal network formation already takes place, as previously reported by our group (Sartore et al., 2017). The combined analyses of the organoids identified and quantified 3,073 proteins with at least two unique peptides (Figure 1B, and data on Supplementary Table 1). Tissue clustering analyses demonstrated enrichment of terms such as fetal brain cortex, epithelium and other brain proteins (Figure 1C). Investigating broad biological processes identified in 45-day cerebral organoids, the proteomic profile analyses showed majorly enriched pathways related to metabolic and cellular processes, such as cell–cell adhesion, RNA splicing, ATP metabolism, cerebral cortex development, and cytoskeleton organization. Specific neuronal functions were also seen, such as glutamate secretion, neuron projection development, axonal transport, and dendrite morphogenesis (Figure 1D). Regarding cellular compartments, proteome analysis comprehended cytosol, membrane, cytoskeleton and nucleoplasm, general cellular compartments, showing a global overview of the cell, in agreement with our whole-cell proteomics approach. Nevertheless, enriched proteins from extracellular exosome, including synaptic vesicle, axon and postsynaptic density were found, in addition to myelin sheath, cell–cell–adherent junction and focal adhesion proteins (Figure 1D).

In addition, using downstream effects analysis of those protein functions, we observe proteins related to cytoskeletal organization with filament bundling, indicating activation of neuronal morphogenesis and neuritogenesis. This included proteins for axon outgrowth, branching, and neurite extension (Figure 1E). On the other hand, the proteins present have ties with inhibition of organismal death, tumorigenesis and apoptosis, while active cell survival, possibly indicating adaptation to organoid culture growth expansion limitations (Figure 1F). These enrichment analyses confirm a global, functional correlation between 45-day whole-brain cerebral organoids and to brain tissue.

Additionally, we compared the proteomes of cerebral organoids with those of fetal human brains with 16–20 GW (Djuric et al., 2017). Djuric et al. (2017) have analyzed the developing human brain in a spatiotemporal manner, specifically the ventricular zone (VZ), intervening intermediate zone (IZ), subplate (SP), and frontal cerebral cortex (Cx). The Spearman correlation of the protein intensities of both datasets [from this study and that of Djuric et al. (2017)], ranged from *r* = 0.28–0.35 (*p* < 1.24E-26), with approximately a 40% overlap of proteins among samples (Supplementary Figure 1A). Because different methodological approaches were applied in each proteomic study, and as information of fetal brain only as young as 16 GW was available; the overlap range is potentially justified, as cerebral organoids were grown for 45 days, and according to previous mRNA correlation studies (Camp et al., 2015; Luo et al., 2016) are expected to correlate to a younger GW. In addition, the overlapping proteins indicate that cerebral organoids exhibit characteristics from all developing cortical layers, equivalently separated into VZ, IZ, SP, and Cx proteins, as the majority of overlapping proteins are common among them (Supplementary Figure 1B). Certain proteins found in common included novel layer markers described by them (Djuric et al., 2017), such as filamin C (FLNC), found mainly in VZ, and cellular retinoic acid binding protein 1 (CRABP1), found in later and more committed neural cells within the VZ during development (Supplementary Table 1). These shared proteins over-represent functions related to cell–cell adhesion, mRNA splicing and translation initiation processes, in addition RNA and protein binding molecules, and found to be principally part of the cytosol, exosome, or myelin sheath compartments (Supplementary Figure 1C).

Additional markers of development found in organoids are from early events, including forebrain dorsal/ventral pattern formation, such as clusterin (CLU), SPARCL1 (SPARC like protein 1) and TTC21B (tetratricopeptide repeat protein 21B); and hindbrain formation, via HOXA1 (homeobox protein Hox-A1). Retinal proteins such as guanylyl cyclase 2 (GUCY2F), dehydrogenase 1 (ALDH1A1), and alpha-crystallin B chain (CRYAB) were also detected (Supplementary Table 1). Evidence of those markers emphasizes the heterogeneity and diversity niches formed by this cerebral organoid model.

We have also compared the cerebral organoid proteomics dataset with that of the BrainSpan fetal transcriptomic dataset^9^, with a focus on the 8–37 post-conception week (dorsolateral prefrontal cortex area). The correlation of RNA-protein is lower than that of protein-protein comparisons, ranging from *r* = 0.02-0.06 (*p* < 0.003) (Supplementary Figure 2A). On the other hand, the comparison of the proteome of cerebral organoids with the RNA-Seq dataset of similar protocols of differentiation of cortical organoids (Camp et al., 2015; Luo et al., 2016), showed a moderate correlation ranged from *r* = 0.19–0.22 (*p* < 5.88E-22), stronger in older organoids (Supplementary Figure 2B). Possible differences are due divergences in translational rates and technical distinctions in mRNA and protein levels during the differentiation process.

### Diversity of Cell Types in Cerebral Organoids

**FIGURE 2:**
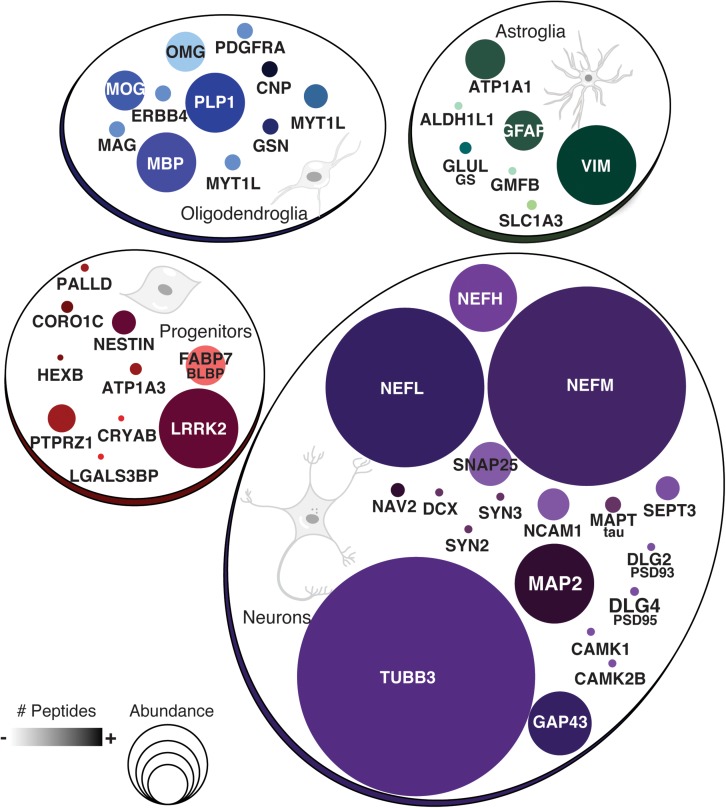
Proteins commonly found in neural cell types. Groups show protein names representative of neurons, astroglia, oligodendroglia, or other progenitor cells. Circle sizes indicate the relative abundance of protein found. Color intensity is equivalent to the number of peptides found per protein.

**FIGURE 3:**
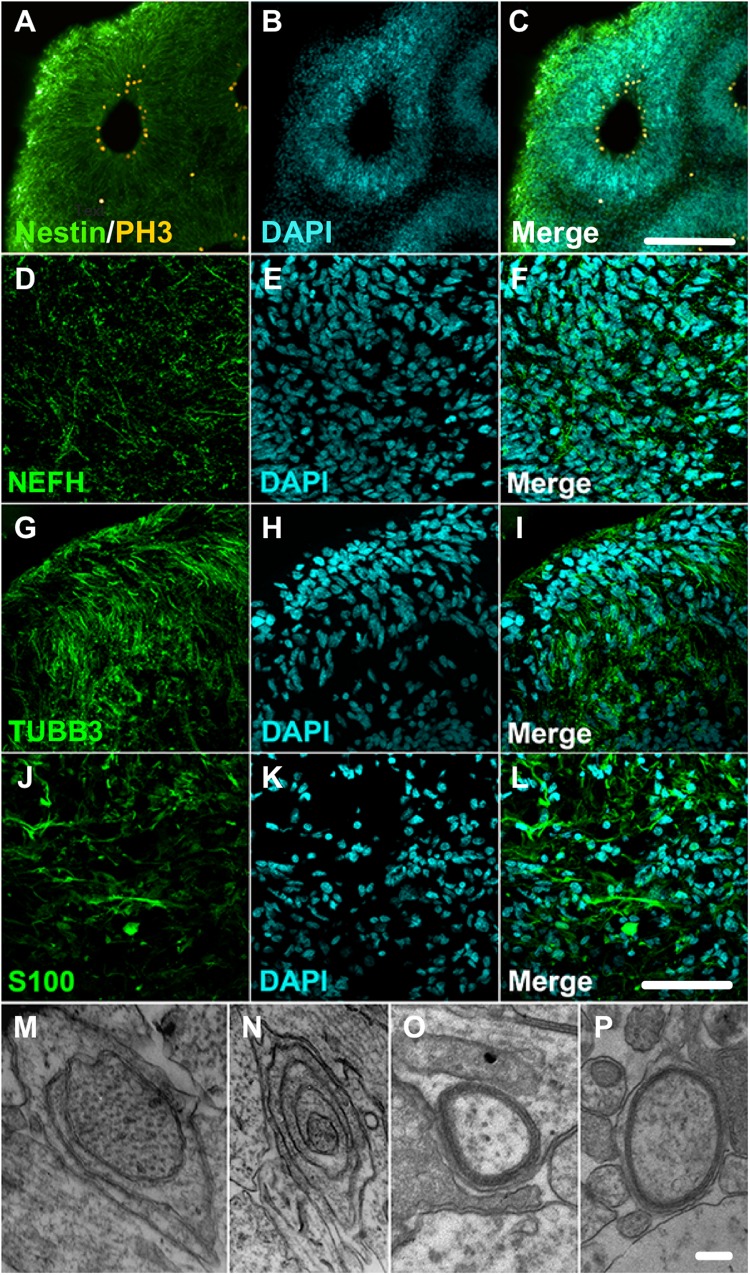
Cerebral organoids expressing neuronal and glial proteins. **(A–C)** Immunostaining of 30-day cerebral organoids with nestin (green) and showing proliferative areas with PH3 (yellow). Immunostaining of 45-day cerebral organoids with neuronal markers **(D–F)** neurofilament 200 (NEFH), and **(G–I)** tubulin βIII (TUBB3), or glial marker **(J–L)** S100 protein in cryosections. Nuclear staining with DAPI. **(M–P)** Transmission electron microscopy images showing myelin sheath formation around some cells of cerebral organoids. Scale bars shown correspond to **(A–C)** 200 μm, **(D–L)** 50 μm, and **(M–P)** 1 μm.

A closer look into the proteome of cerebral organoids reveals specific proteins from a wide range of neural cell types related to neurodevelopment. Overall, Figure 2 (along with Supplementary Table 1) depicts how broad the protein dynamics are, and highly abundant cytoskeleton proteins can be observed, such as nestin (NES), tubulin β3 (TUBB3), neurofilaments (NEFL, NEFM, NEFH), microtubule-associated protein 2 (MAP2), and microtubule-associated protein tau (MAPT), and neural cell adhesion molecule 1 (NCAM1). Low-abundance proteins however were more specific to differentiation processes and other neuron-related proteins. This included sodium/potassium-transporting ATPase subunit alpha-3 (ATP1A3), synaptosomal associated protein 25 (SNAP25), and leucine-rich repeat kinase 2 (LRRK2), indicating the presence of both progenitor and neuronal cells. Several of these neural proteins are commonly found in proteomes of human brain tissue (Carlyle et al., 2017). The presence of these neural proteins is widespread in cerebral organoids. Cell proliferation zones are shown in yellow by phospho-H3 protein (PH3), surrounding ventricle-like cavities, and are surrounded by nestin-expressing cells (Figures 3A–C). Other neuronal cytoskeleton proteins were found to be widespread throughout cerebral organoids, such as the neurofilament heavy (NEFH, in Figures 3D–F), an intermediate filament polypeptide, and the neuronal microtubule protein tubulin (TUBB3, Figures 3G–I).

The proteomic analysis of cerebral organoids has also unveiled proteins associated with glial cell types, such as glial fibrillary acidic protein (GFAP), vimentin (VIM), glia maturation factor beta (GMFB), and aldehyde dehydrogenase 1 family member L1 (ALDH1L1). Those proteins are commonly found in astrocytes, and represented here by the S100 protein (Figures 3J–L); several astroglial cells were found in the cerebral organoids. Consistently, though to a lesser extent, proteins related to an oligodendroglial phenotype were also present. Which included not only proteins related to OPC stage, such as 2′,3′-cyclic nucleotide 3′phosphodiesterase (CNP) and platelet-derived growth factor receptor alpha (PDGFRA), but also proteins related to more mature oligodendrocyte transitional stages, such as myelin basic protein (MBP), myelin proteolipid protein (PLP1), and myelin oligodendrocyte glycoprotein (MOG). Indeed, although early in development and variable within organoid formation, specific niche environments allow some organoid areas to present initial stages of myelin sheath formation around neurite bundles, in early stages of lamellae formation, as observed in transmission electron microscopy (Figures 3M–P). Additionally, myelinating proteins were among those correlated with fetal brains (16–26 weeks) (Djuric et al., 2017) despite the completed process being associated with later developmental stages. According to this data, cerebral organoids are composed mainly of progenitors, including radial glia, and neurons, followed by astroglia and, to a lesser extent, oligodendroglia.

### Isolation and Maturation of OPCs From Cerebral Organoids

**FIGURE 4:**
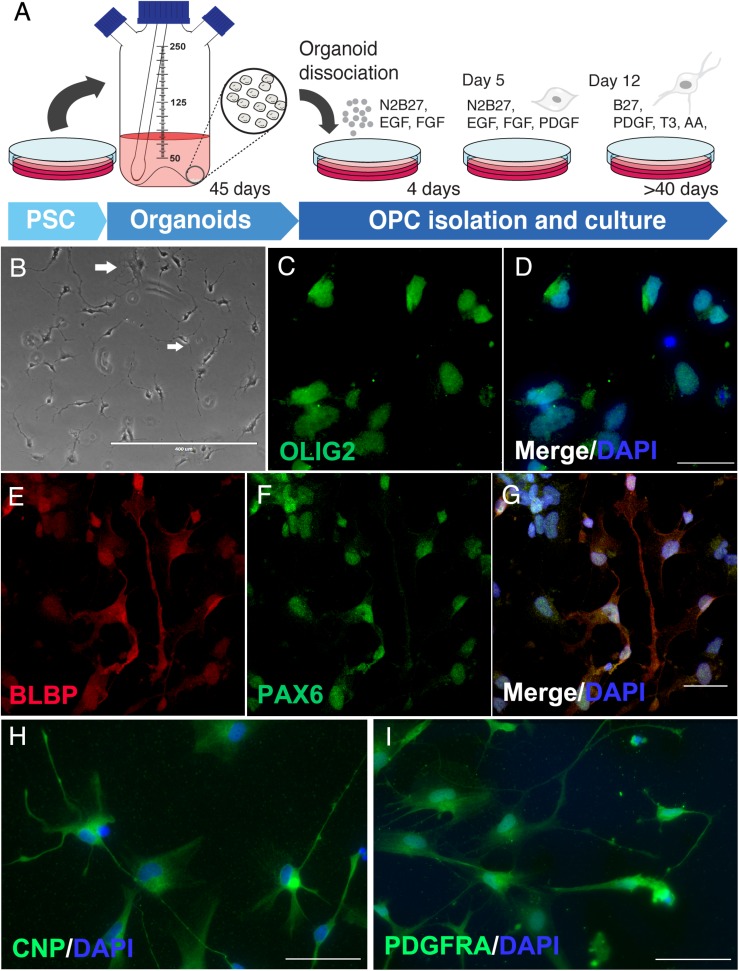
Oligodendrocyte progenitors derived from cerebral organoids. **(A)** Schematic representation of OPC isolation and culture. **(B)** Phase contrast of immature oligodendrocytes with initial ramifications (arrows in white). OPCs were stained for **(C,D)** olig2, oligodendrocyte lineage transcription factor, **(E–G)** BLBP and PAX6 progenitor markers, **(H)** CNPase (CNP), enzyme of early oligodendrocyte formation and myelin precursor, and **(I)** PDGFRA oligodendrocyte lineage marker. Scale bars shown are **(B)** 400 μm, **(D,G)** 30 μm and **(H,I)** 50 μm.

Due to the presence of oligodendroglial proteins, we hypothesized the possibility of isolating human oligodendrocytes from cerebral organoid cultures. Following a similar approach as previously described for astrocytes (Dezonne et al., 2017), an isolation protocol was developed to extract progenitors, including OPCs from those 45-day cerebral organoids and was followed by 2D maturation *in vitro*. After proliferative progenitors were isolated from the whole organoid, OPC cultures were further differentiated for up to 60 days (Figure 4A). For the length of the culture period, cells with a bipolar morphology composed the majority of OPCs, while some had initiated active cytoplasm ramification toward a multipolar morphology, typical of more mature intermediate oligodendrocytes (Figure 4B, arrows). These OPCs in culture, bipolar or multipolar, were labeled with radial glia markers BLBP and PAX6 (Figures 4E–G), and early event markers of oligodendrogenesis such as OLIG2, CNPase (CNP) and PDGFRA (Figures 4C,D,H,I).

### Calcium Signaling Analysis of Neural Cells in PSC-Derived Cerebral Organoids

Based on the identification of proteins associated with axon guidance and neurotransmitter secretion, there is indication of the presence of maturing neurons in cerebral organoids. Here, we used calcium transient imaging to measure neuronal activity of cultured cerebral organoid cells. These neural cells are physiologically balanced by neurotransmitter exocytosis, which is constantly modulated by the levels of intracellular calcium [Ca^2+^]i. Proteomic data have positively identified Cav1.2 (CACNA1C – voltage-dependent L-type calcium channel subunit alpha-1C), in addition to the regulatory subunit CACNA2D3 (voltage-dependent calcium channel subunit alpha-2/delta-3) (Supplementary Table 1).

**FIGURE 5:**
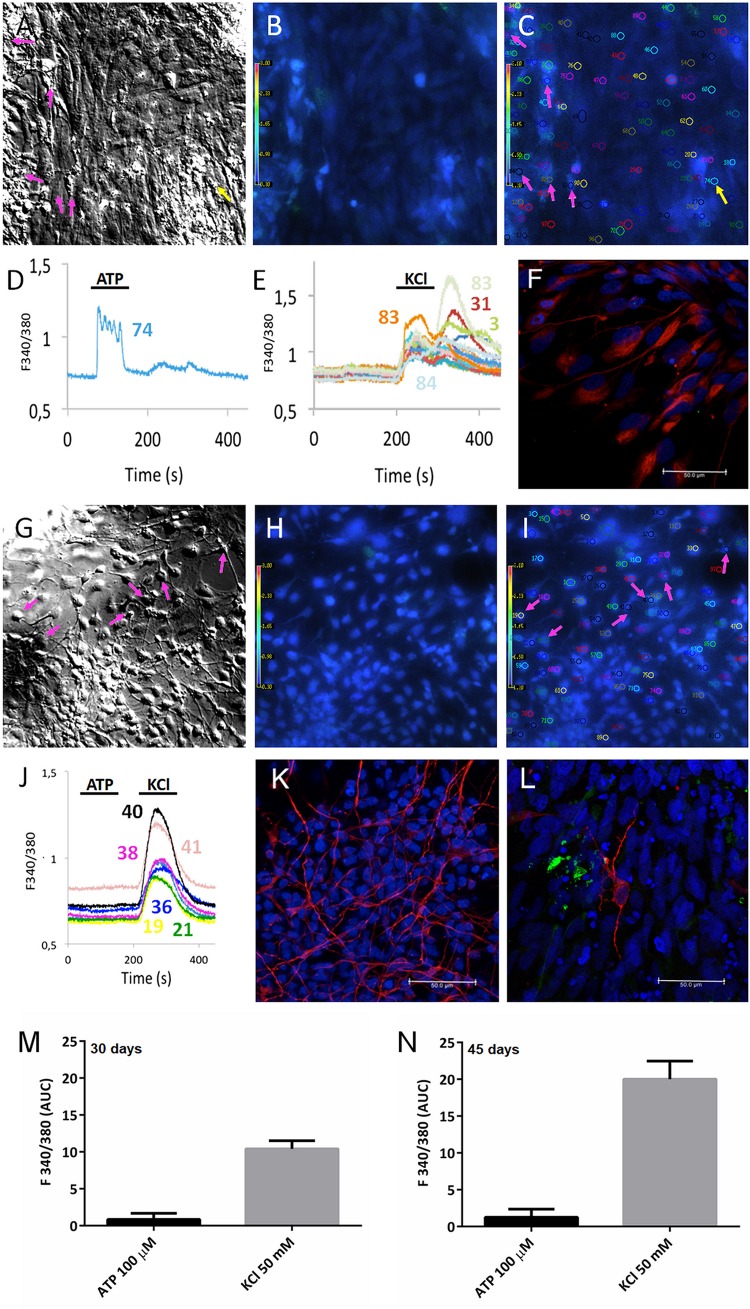
Calcium response to ATP and KCl stimulation of cerebral organoids. PSC-derived cerebral organoids grown for **(A–C)** 30 or **(G–I)** 45 days had their cells dissociated and plated for 10 days on 15 mm coverslips. Cultured cells shown in **(A,G)** bright field and **(B,H)** under fluorescence. **(C,I)** Selected cells are shown in the same microscope field under fura-2 fluorescence in SCCI experiments. Typical responses are shown for cells when stimulated with **(D)** 100 μM ATP or **(E,J)** 50 mM KCl. As shown in **(C–E)**, only one cell (#74) responded exclusively to ATP (yellow arrow), whereas several cells (# 3, 31, 82, 83, 84) responded to KCl (pink arrows), in 30-day cerebral organoids. As shown in **(I,J)** selected cells (# 21, 36, 38, 40, and 41) were only responsive to 50 mM KCl (pink arrows) in 45-day cerebral organoids. Post-stimulation, coverslips were fixed and cells labeled for **(F)** nestin, **(K)** TUJ1/tubulin βIII (TUBB3), or MAP2 **(L)** scale bars 50 μm. Areas under the curve are representative of calcium responses to ATP or KCl obtained from 30-day **(M)** or 45-day organoids **(N)**.

Dissociated human cerebral organoids displayed more flat cells (Figure 5A) or neuron-like cells (Figure 5G). Their equivalent fluorescence microscopic fields are shown in Figures 5B,H, respectively. Additionally, the numbers illustrated in Figures 5C,I Fura-2 fluorescence of selected cells, in the same microscopic fields, are regions of interest selected before stimulation with 50 mM KCl or 100 μM ATP; then, single cell calcium variations were evaluated by quantifying the ratio of the fluorescence and later matched with the cell number, showing that most of the responses (cells # 3, 31, 82, 83, 84 were activated by KCl, pink arrows in Figure 5E), but not by ATP. On the other hand, a flat-like cell (#74) – identified by a yellow arrow – is activated by 100 μM ATP (Figure 5D) but not by KCl. Several of those cells were labeled by nestin (Figure 5F) and TUBB3 (Figure 5K), with a few MAP2 positive cells (Figure 5L), suggesting they are progenitors and/or neuronal-like cells, which respond to 50 mM KCl (Figure 5E). As the organoids mature, coverslips presented neuron-like cells (for example, cells # 21, 36, 38, 40, and 41) and were only responsive to KCl (Figure 5J), suggesting those cells express voltage dependent calcium channels. In total, 607 cells were analyzed from 30- and 45-day cerebral organoids, in which 245 cells (40%) were responsive to 50 mM KCl, while only 5 cells (1%) were activated by 100 μM ATP. The area under the curve was quantified for those cultured flat cells (Figures 5A,M), or neuron-like cells (Figures 5G,N), which responded to 100 μM ATP or 50 mM KCl, as previously done (Freitas et al., 2019). This percentage agrees with the previously addressed protein content, which indicated the presence of more neuronal than glial cells in organoids at this differentiation stage, several of which are young and still unresponsive.

### Protein Signals Involved With Neuronal Patterning of Cerebral Organoids

**FIGURE 6:**
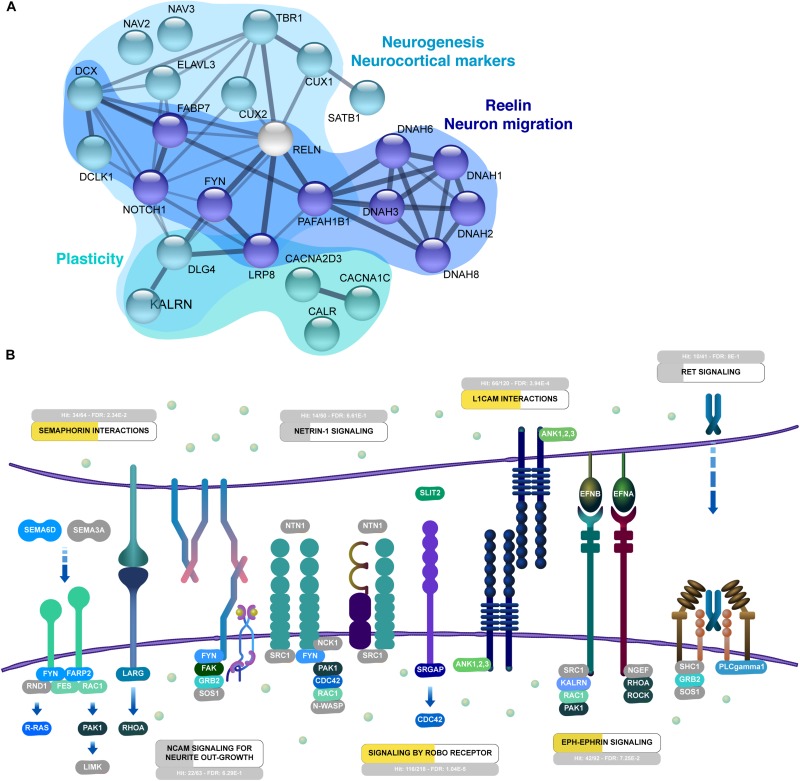
Interactive organization of proteins from 45-day cerebral organoids. **(A)** Network representation of selected proteins related to neurogenesis, reelin and migration of neurons, and synaptogenesis. Networks are based on the STRING database (https://string-db.org/). **(B)** Neuronal guidance is overrepresented in 45-day cerebral organoids (FDR 2.05E-15). Enriched signaling pathways include semaphorin interactions (FDR 5.14E-4), L1CAM interactions (FDR 9.68E-7), signaling by ROBO receptor (FDR 4.9E-9) and Eph-Ephrin signaling (FDR 3.6E-3). Proteins in NCAM signaling for neurite outgrowth, netrin-1, and ret signaling are partially represented. Overrepresentation analyses were performed with Reactome (https://reactome.org/). Proteins found in proteomics analyses of 45-day organoids are colored in shades of blue and green, while gray proteins were not found in the dataset.

Cells in the differentiating cerebral organoids followed patterns seen in the nervous system to acquire distinct identities. Among the neuronal patterning proteins found in cerebral organoids, we observed downstream reelin pathway proteins (Figure 6A), such as FABP7/BLBP (fatty acid binding protein 7, also known as brain lipid-binding protein), and PAFAH1B1 (platelet activating factor acetyl hydrolase 1b regulatory subunit 1, also known as LIS1 - lissencephaly 1 protein), in addition to the surface receptor LRP8 (Low-density lipoprotein receptor-related protein 8, also known as ApoER2 – apolipoprotein E receptor 2) and dyneins (DNAH1, 2, 3, 6, 8). This pathway is triggered by activation of NOTCH1 (neurogenic locus notch homolog protein 1) and FYN (tyrosine-protein kinase Fyn), which were also detected in cerebral organoids (Figure 6A).

These proteins are connected to protein markers of neurogenesis and patterning, including ELAVL3 (ELAV like RNA binding protein 3, also known as HuC – Hu antigen C and paralog to HuD, neuronal-specific RNA-binding proteins), and the special AT-rich DNA-binding protein 1 (SATB1), which plays regulatory roles in neuronal differentiation (Figure 6A). In addition, proteins associated with the radial glia scaffold were also present in organoids (Figure 6A), such as doublecortin (DCX), DCLK1 (doublecortin-like kinase 1/Serine/threonine-protein kinase DCLK1), the neocortical markers CUX1 and CUX2 (homeobox protein cut-like 1 and 2, respectively), TBR1 (T-box brain protein 1), and neuronal navigator proteins 2 and 3 (NAV2 and NAV3).

These findings indicate plasticity of the model, supported by the presence of calcium-binding protein calretinin (CALB2) in cerebral organoids, a protein that is abundantly found in neurons of the retina and cortical interneurons. This is in addition to the presence of other signaling proteins from the calcium-calmodulin family, such as the calcium/calmodulin-dependent protein kinase type I (CAMK1), type II subunit alpha (CAMK2A), and type II subunit beta (CAMK2B) (Figure 6A). Overall, this reflects the different levels cell signaling pathways we are able to capture, and its similarities to the neural developmental *in vivo*.

### Development of Axonal Guidance in Cerebral Organoids

The proteins identified in cerebral organoids show an overrepresentation of pathways related to axonal guidance, which includes semaphorin (FDR 2.34E-2), L1CAM signaling (FDR 3.9E-4), Robo receptor signaling (FDR 1.04E-5) and Eph-Ephrin signaling (FDR 7.25E-2) (Figure 6B). In regards to semaphorins, protein SEMA6D and the signaling cascade with FYN, FARP2 (FERM_RhoGEF and pleckstrin domain-containing protein 2), and RAC1 (Ras-related C3 botulinum toxin substrate 1) were found (Figure 6B). These are common interactors of the CRMP/DPYSL protein family (collapsin response mediator protein/dihydropyrimidinase like), such as DPYSL2-5, which are related to microtubule assembly and growth cone collapse (Supplementary Figure 3). Several of the secretory and membrane proteins that are related to semaphorin, netrin, or ephrin signaling are connected to extracellular matrix (ECM) proteins, such as laminin (LAMA1, A3, A4, A5, B1, C1), fibronectin (FN1), and proteoglycans (i.e., basement membrane-specific heparan sulfate proteoglycan core protein - HSPG2). The ECM regulates neuroepithelial growth, signaling via cell-surface integrins alpha-6 and beta-1 (ITGA6 and ITGB1), proteins supporting interconnection of cells in the organoids (Supplementary Figure 3).

Regarding pathways controlled by cell adhesion molecules, the L1CAM signaling pathway has possible molecular interactions with ankyrins (ANK1, 2, 3), proteins that play key roles in neurite extension and inter-neuronal adhesion (Supplementary Figure 3). Signaling by Robo Receptor – another key developmental axonal guidance pathway, which regulates cell migration, proliferation, and intermediate progenitor transition – is represented by ROBO1 (roundabout homolog 1). It could signal via SRGAP1 and 3 (SLIT-ROBO Rho GTPase-activating protein 1 and 3) and CDC42BPB (CDC42 binding protein kinase beta) (Figure 6B). Slit-Robo signaling via netrin-1/DCC on a ROBO1-DCC interaction (netrin receptor DCC) is necessary for appropriate neuronal maturation. Afterward, the Eph-Ephrin pathway, via expression of ephrin (EFNB1) and its preferred receptors EPHB3 and EPHB1, could lead to axonal orientation and subsequent synaptic maturation (Figure 6B). EPHB2, EPHA3, and EPHA8, other receptors found in the organoids, are known to regulate dendritic spine development, along with a plethora of signaling molecules, including kalirin (KALRN), RAC1 (Ras-related C3 botulinum toxin substrate 1), and PAK1 (serine/threonine-protein kinase PAK 1) (Figure 6B).

### Neuronal Subtypes and Synaptogenesis Starting in Cerebral Organoids

**FIGURE 7:**
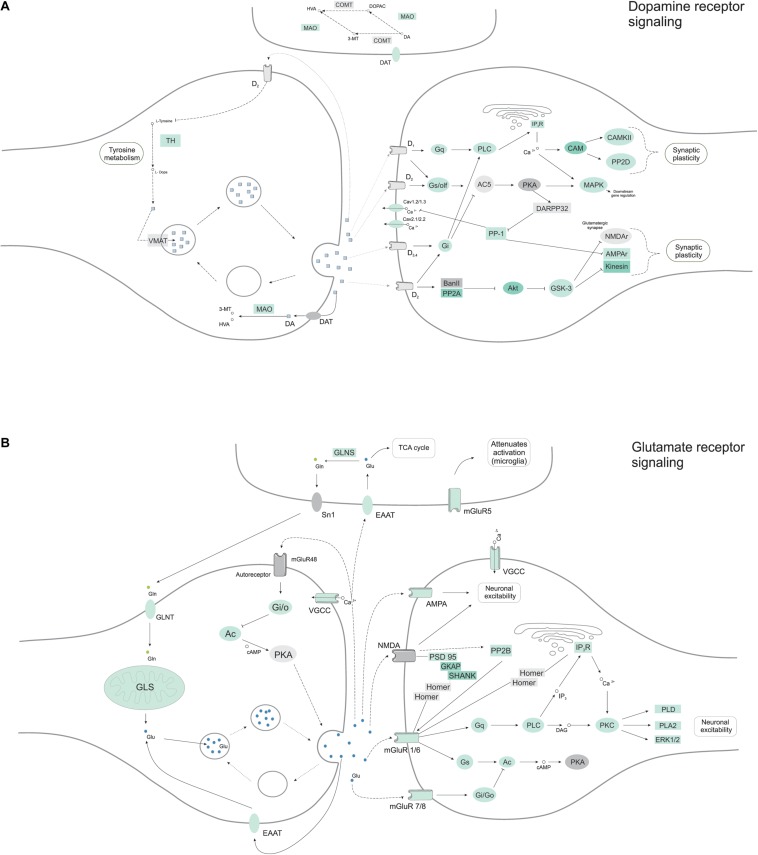
Neuronal molecular pathways indicate glutamate and dopamine receptor signaling, in a representation of 45-day cerebral organoid proteomes. Pathway interactions of **(A)** dopamine and **(B)** glutamate receptor signaling were analyzed from the proteome dataset using the KEGG database (http://www.kegg.jp/). Proteins found in proteomic analyses are colored in green, while gray proteins were not found in the dataset.

Having seen protein networks related to axon guidance and positioning, and previous indications of functional neurons like calcium signaling, we expected to find a diversity of neuronal subtypes. Grouping proteins by their transmission function (glutamatergic, dopaminergic, serotonergic or GABAergic neurons), we found proteins related to two main types of neurons (Figure 7) in cerebral organoids. Proteins belonging to dopaminergic (*p*-value 1.5E-3) and glutamatergic (*p*-value 1.3E-2) synapse were predominantly enriched within the set of proteins found in the cerebral organoids. Around 41 proteins from the dopamine receptor signaling were found (Figure 7A and Supplementary Table 2), among them tyrosine hydroxylase (TH, tyrosine 3-monooxygenase), a marker for dopaminergic neurons, and calcium/calmodulin dependent protein kinase II alpha (CAMK2A), a key protein that stimulated dopamine efflux. Post-synaptic signaling molecules, like PSD-95 and SHANK1 (SH3 and multiple ankyrin repeat domains protein 1), were found, supporting the presence of synaptogenesis. And 34 proteins related to the glutamate receptor signaling (Figure 7B and Supplementary Table 2), including glutamate receptor 4 (GRIA4), a subunit present on AMPA receptors, and others such as metabotropic glutamate receptor 8 (GRIM8, mGluR8). We also identified 17 proteins related to GABAergic synapse, such as CALB2, which is usually found in GABAergic interneurons, as previously mentioned, was also found, however, this pathway was not significantly enriched (*p* > 0.05).

## Discussion

Cerebral organoids have emerged as an unparalleled functional model of the human brain, allowing this organ to be studied mainly during neurodevelopmental stages (Lancaster and Knoblich, 2014; Quadrato and Arlotta, 2017). Previous studies (Lancaster et al., 2013, 2017; Qian et al., 2016; Bershteyn et al., 2017; Birey et al., 2017), including from our group (Sartore et al., 2017), have shown that cerebral organoids are self-organizing and that regionalization follows several neurodevelopmental steps, with a variety of heterogeneous micro-niches being present (Renner et al., 2017). Due to extensive cellular variability, self-patterning, and various tissue architectures, more functional neural networks can be achieved (Quadrato and Arlotta, 2017). Pluripotent stem cell-derived cerebral organoids are still an under-development tool, holding great promise in future studies in the quest of understanding the complexity of the human brain cellular network (Paşca, 2018). Therefore, our effort was to unfold that cell diversity and molecular complexity by investigating the proteome of PSC-derived organoids, providing snapshots of the early developmental stages of neural protein networks, using an unbiased method.

The main efforts to study organoids has been to uncover the typical cellular compositions of neurodevelopmental stages (Lancaster et al., 2013; Camp et al., 2015; Quadrato et al., 2017; Velasco et al., 2019). Using a previously described protocol for whole-cortical organoid (Lancaster et al., 2013; Sartore et al., 2017), cerebral organoids were differentiated for 45 days from human pluripotent stem cells (hPSC) to access their proteome content. At this stage, cerebral organoids present a well-organized structure with ventricle-like cavities, contained cell proliferation zones, and expressed intermediate progenitors followed by early differentiation stage neurons (Sartore et al., 2017). Therefore, while the cytoarchitecture of cerebral organoids has been shown to be complex (Quadrato et al., 2017; Renner et al., 2017), and contains several cell types, when it comes to studies of molecular behavior, there is still much to be discussed. The developmental cues of corticogenesis, including those of a radial glia, are present in the proteome of cerebral organoids. Immature neurons, regulated by the reelin (RELN) signaling pathway, are able to migrate, position, and establish cortical layers to support early synaptic circuits, laminar organization, and further interconnections (Bystron et al., 2006; Sekine et al., 2012). Despite RELN itself not being found in our dataset, downstream proteins of its pathway were present and might contribute to patterning. Several other key developmental pathways of cell migration, proliferation, and axonal guidance follow these signals. Such as Robo receptor signaling, which confers transition of primary to intermediate progenitor via ROBO1 (Borrell and Götz, 2014); or markers such as HuC and SATB1. Moreover, L1CAM signaling pathway participates in the projection neurons crossing the brain midline (Demyanenko et al., 1999), to avoid aberrant axonal trajectories. In addition to neuronal navigator proteins 2 and 3 (NAV2 and NAV3, respectively), proteins previously shown to be connected to neurite outgrowth and axonal elongation upon interaction with the vitamin A metabolite all-trans retinoic acid (atRA) (Merrill et al., 2002; Muley et al., 2008). All of which implicate the organoids into setting early migrating environment, axonal outgrowth, and prone for synapses formation.

The protein–protein interactions observed here indicate intricate mechanisms involved in the migration of progenitor cells, radial glia, or oligodendrocyte progenitors following the neural program. This state-of-the-art proteomics data provided a broader assortment of the prevailing proteins in cerebral organoids, and complements previous studies that have highlighted global and single-cell RNA expression (Camp et al., 2015; Bershteyn et al., 2017; Quadrato et al., 2017). Correlating to protein expression of the human brain, the 45-day-old, whole-brain cerebral organoids depicted representative brain proteins, and a reasonable similarity with *in vivo* human cortex development, shown by revealing their functionality and connecting pathways among those different cell types, already at the early stages. A recent study showed the same similarity between cell-type patterns found in organoids and fetal human brain by comparing scRNA-seq dataset, with about 13% glial cells in 3-month old organoids (Velasco et al., 2019). Glial cells have roles in several steps of remodeling and consolidation, in addition to a carefully orchestrated regulation of transcription factor expression (as reviewed in Allen and Eroglu, 2017). Some of the pathways found overrepresented in cerebral organoids, and which participate in the regulation of neural differentiation, coincide with the genesis of different neuronal cell types and glia cells via cell-to-cell interactions. These include glypican signaling, SPARCL1/hevin and thrompospondin (THBS1), promoting synaptogenesis (Allen and Eroglu, 2017). Several of the cell surface receptors that were found, such as the tyrosine phosphatase family PTPRs, including PTPRD and PTPRS (receptor-type tyrosine-protein phosphatase delta and S), are required for a normal brain development (Coles et al., 2011). They have roles in the pre- and post-synaptic differentiation of neurons, neurite outgrowth driven by ECM stimuli, and glial cell-secreted factors. For instance, PTPRZ1 (receptor-type tyrosine-protein phosphatase zeta) and contactin-1 (CNTN1), which modulates outer radial glia and oligodendrocyte progenitor proliferation, are responsible for maintenance of the stem cell niche (Pollen et al., 2015), and switch to myelinating mature cells to insulate neurons (Lamprianou et al., 2011), and have not been explored.

Most studies of human brain proteomics have been performed on adult postmortem tissue, with only a few on the developing brain, then being predominantly postnatal brain tissue (Carlyle et al., 2017; Djuric et al., 2017). Despite cerebral organoids being a somewhat simpler model of the fetal human brain, it can be adequate to recapitulate several events following directional developmental cues. Additionally, it is an adequate model to study signaling events that are dependent on particular cellular niches within the organoid. Containing separate neuroepithelial layers, the organoids are comprised of a structure similar to cerebral ventricles, with a possible separation of ventricular and subventricular zones, in common with early human brain development. This have been previously shown by the genetic program of previous studies of differentiating organoids (Camp et al., 2015; Luo et al., 2016; Quadrato et al., 2017; Velasco et al., 2019). Our approach revealed major functional proteins expressed at one specific moment; yet, several transcription factors that are often found throughout neurodevelopment – and which are considered markers of regionalization and cell type specification – might not be detected using this total-cell, whole-cerebral organoid, due to their low-abundance and/or discovery limitations inherent to the method. Regardless, this provides a broader understanding of the signaling pathways that are present, consequently predicting phenotypical outcomes.

In accordance with early stages of development, following an orderly formation of neurons and layers, we observe proteins for a proliferative pool of progenitors, neuronal development and the presence of glutamatergic and dopaminergic neuronal subtypes. Those early events indicate functionality at this stage, with signaling processes that regulate migration of neuronal cells, involving changes in Ca^2+^ concentration. As shown here, several of the proteins found in cerebral organoids indicated migration. Others, such as calcium-binding protein calretinin, abundantly found in neurons of the retina and cortical interneurons, or the calcium-calmodulin family, suggest activation of migration, plasticity, and regulation of dendritic spines in cerebral organoids, via NMDA receptor Ca^2+^ influx signaling, when interacting with PSD-95/DLG4 (disk large homolog 4) (Wayman et al., 2008). Neuronal migration has been highlighted by other studies using organoids as a model for neurodevelopment (Quadrato et al., 2017; Renner et al., 2017).

Additionally, as presented here, whole-brain organoids have the potential for gliogenesis and oligodendrogenesis. On a lower level, proteins involved in the switch of producing neuronal cells to glial precursors are present, including GFAP, MBP, and PLP, despite the early stage in development. We have previously shown functional astrocytes derived from glial precursors of cerebral organoids (Dezonne et al., 2017). Here, we demonstrated that CNPase expressing OPCs could be derived from those organoids in culture, providing, at the least, a potential for testing OPC differentiation *in vitro.* In addition, further development of organoids using growth factor specific-stimulation could lead to more diverse subtypes of glial cells, including myelinating oligodendrocytes (Paşca et al., 2015; Bershteyn et al., 2017; Iefremova et al., 2017; Quadrato et al., 2017; Madhavan et al., 2018).

More recently, several groups aimed for more regionalized or cell-specific organoids to understand development and disease modeling. Comprised of particular morphogens and growth factors for patterning, organoids can achieve a more specific regionalization, such as midbrain-like (Jo et al., 2016), and hypothalamus organoids (Qian et al., 2016), fusion of cortical to medial ganglionic eminence organoids (Xiang et al., 2017), and cortico-thalamic fused organoids (Bagley et al., 2017). Several studies have pursued an enrichment of cell types, such as long-term cerebral organoid cultures, increasing astrocyte (Paşca et al., 2015; Sloan et al., 2017), oligodendrocyte (Marton et al., 2019), and microglial cell (Ormel et al., 2018) production within organoids, or achieving neuronal maturation able to achieve muscle contraction (Giandomenico et al., 2019). More homogeneous and reproducible spheroids have also been a modeling idea aiming on cell diversity for drug testing development (Pamies et al., 2017; Madhavan et al., 2018; Velasco et al., 2019). This ultimate opens new paths to better understand and model the human brain.

## Conclusion

We provide a large-scale protein level evidence of neurodevelopmental processes and pathways occurring within the *in vitro* cerebral organoid model. Here, we have shown some of the main protein interactions related to neocortical development, including those related to proliferation and cell-fate specification, axonal guidance, synapse formation, and beginnings of myelin sheath formation. This provides evidence of the heterogeneity of this model and the robustness of a whole-organoid proteomics approach. We understand that our samples sizes are modest, and that future studies with higher throughput over an expanded time course would be valuable. However, global representation of protein families and networks in cerebral organoids are still scarce. Much of the knowledge presented here validates the organoid model beyond the transcriptional level, as adequate to investigate several brain networks. Proteome-wide information can be particularly important to study human brain development and how diseases might develop when pathways are disrupted. Currently, the challenges are toward developing differentiation strategies to yield disease-relevant organoids and producing selective subpopulations of glial and neuronal cell types in later stages of maturation to address more direct questions. Combining proteomics with recent advances in human PSC-derived organoids widens the possibilities to test different hypotheses, several of which have been generated exclusively from post-mortem brain studies. Specific pathways could also increase translational targets in a protein-driven approach for drug-development and human disease modeling.

## Data Availability Statement

The proteomics datasets generated for this study can be found in the PRIDE proteomics data repository (https://www.ebi.ac.uk/pride/archive/) with the accession number PXD011605.

## Author Contributions

JN, DM-D-S, and SR contributed to the conception and design of the study. JN, VS-C, RS, RMC, CS, HF, MM, and RMR performed the experiments, and collected, assembled, and analyzed the data. JN wrote the first draft of the manuscript. VS-C, RS, RMC, HF, and RMR contributed to the writing of the manuscript. JN, SR, and DM-D-S reviewed the final manuscript. All authors approved the final version of the manuscript.

## Conflict of Interest

MM was employed by Waters Corporation. The remaining authors declare that the research was conducted in the absence of any commercial or financial relationships that could be construed as a potential conflict of interest.

## Footnotes

1 https://david.ncifcrf.gov/

2 http://www.pantherdb.org/

3 https://geneanalytics.genecards.org/

4 https://discovery.lifemapsc.com/

5 https://reactome.org/

6 http://www.kegg.jp/

7 https://string-db.org/

8 http://www.brainspan.org/

9 http://www.brainspan.org/
