# Supplementary material for: Human Cerebral Organoids and Fetal Brain Tissue Share Proteomic Similarities
Source: Front Cell Dev Biol. 2019 Nov 28;7:303. doi: 10.3389/fcell.2019.00303 (PMC6893972; doi:10.3389/fcell.2019.00303)
Supplement: Supplementary file 1 [file Data_Sheet_1.pdf]

Human cerebral organoids and fetal brain tissue share proteomic similarities: enabling alternative models to study psychiatric disorders

Juliana Minardi Nascimento[1,2], Verônica M Saia-Cereda[1], Rafaela C. Sartore[2,3], Rodrigo Madeiro da Costa[2], Clarissa S. Schitine[4,5], Hercules Rezende Freitas[5,6], Michael Murgu[7], Ricardo A. de Melo Reis[5], Stevens K Rehen[2,4*], Daniel Martins-de-Souza[1,8,9*]

Supplementary information

Supplementary Table 1: Protein expression of PSC-derived, 45-day cerebral organoids.

(Excel file)

Supplementary Table 2: Enriched proteins of glutamatergic and dopaminergic synapse

(Excel file)

Supplementary Figure 1, 2 and 3

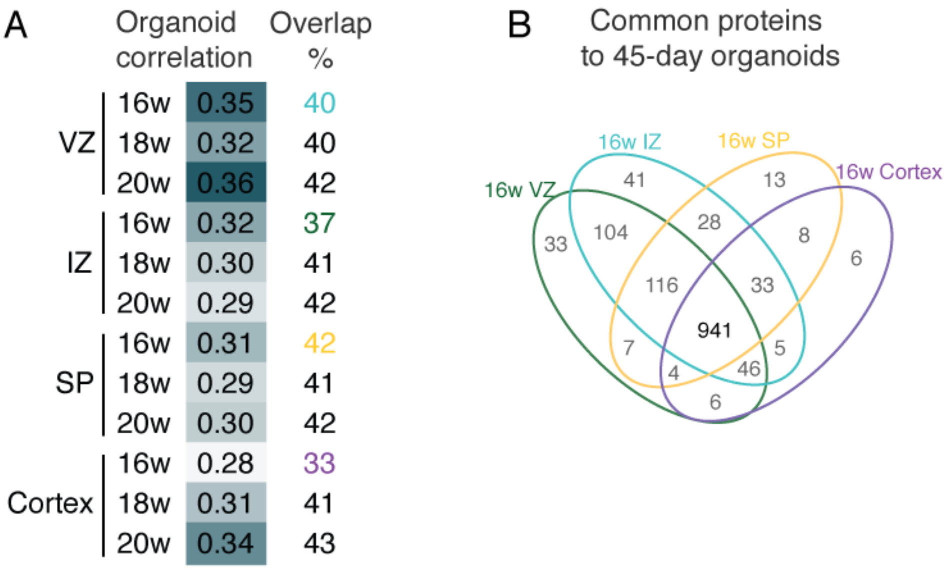

C Common proteins of 16 week VZ, IZ, SP and cortex to organoids

	GO Terms	FDR
**Biological Processes**	Cell-cell adhesion	1.22E-40
	mRNA splicing	5.84E-28
	Regulation of mRNA stability	9.71E-28
	Translational initiation	2.62E-23
**Cellular Compartment**	Extracellular exosome	1.48E-162
	Cytosol	3.26E-110
	Myelin sheath	7.03E-68
	Membrane	1.59E-60
**Molecular Function**	Poly(A) RNA binding	1.69E-109
	Protein binding	1.70E-58
	Cadherin binding involved in cell-cell adhesion	1.17E-45
	RNA binding	1.02E-36

SUPPLEMENTARY FIGURE 1: Comparison of cerebral organoid proteomes with developing brain sub-compartments (ventricular zone, VZ; intermediate zone, IZ; subplate, SP; cortex). **(A)** Spearman correlation of 45-day cerebral organoid proteomes to 16-20 weeks (VZ, IZ, SP, and cortex proteomes). **(B)** Venn diagram of proteins commonly found in 45-day cerebral organoids and 16-week developing human brain areas. **(C)** Gene ontology of the proteins found in common to the 16-week (VZ, IZ, SP, and cortex) proteomes and cerebral organoids proteomes.

A

		Fetal (DFC) post conception week (pcw)										
	Proteomics 45d_Organoids	8_pcw	9_pcw	12_pcw	13_pcw	16_pcw	17_pcw	19_pcw	21_pcw	24_pcw	26_pcw	37_pcw
Proteomics 45d_Organoids	1.00	0.06	0.03	0.02	0.02	0.04	0.02	0.02	0.02	0.03	0.05	0.02
Fetal (DFC) 8_pcw	0.06	1.00	0.92	0.84	0.83	0.87	0.79	0.82	0.81	0.82	0.72	0.76
9_pcw	0.03	0.92	1.00	0.85	0.90	0.85	0.85	0.85	0.85	0.84	0.73	0.78
12_pcw	0.02	0.84	0.85	1.00	0.95	0.94	0.90	0.89	0.90	0.92	0.70	0.79
13_pcw	0.02	0.83	0.90	0.95	1.00	0.92	0.95	0.94	0.93	0.93	0.74	0.84
16_pcw	0.04	0.87	0.85	0.94	0.92	1.00	0.91	0.91	0.92	0.96	0.74	0.83
17_pcw	0.02	0.79	0.85	0.90	0.95	0.91	1.00	0.99	0.97	0.95	0.76	0.88
19_pcw	0.02	0.82	0.85	0.89	0.94	0.91	0.99	1.00	0.97	0.94	0.78	0.89
21_pcw	0.02	0.81	0.85	0.90	0.93	0.92	0.97	0.97	1.00	0.96	0.82	0.93
24_pcw	0.03	0.82	0.84	0.92	0.93	0.96	0.95	0.94	0.96	1.00	0.79	0.91
26_pcw	0.05	0.72	0.73	0.70	0.74	0.74	0.76	0.78	0.82	0.79	1.00	0.88
37_pcw	0.02	0.76	0.78	0.79	0.84	0.83	0.88	0.89	0.93	0.91	0.88	1.00

Colour scale: 1.0, 0.5, 0, -0.5, -1.0

B

		human Organoids (hOrg) Camp et al. 2015							Cortical organoids (CO) Luo et al. 2016		
	Proteomics 45d_Organoids	hOrg_33d	hOrg_35d	hOrg_37d	hOrg_41d	hOrg_53d	hOrg_58d	hOrg_65d	CO_40d	CO_60d	Fetal_Ctx
Proteomics 45d_Organoids	1.00	0.18	0.18	0.18	0.19	0.19	0.21	0.20	0.22	0.22	0.20
Camp et al. 2015 hOrg_33d	0.18	1.00	0.98	0.98	0.94	0.94	0.94	0.94	0.65	0.63	0.48
hOrg_35d	0.18	0.98	1.00	0.98	0.93	0.94	0.94	0.94	0.66	0.64	0.49
hOrg_37d	0.18	0.98	0.98	1.00	0.95	0.94	0.94	0.95	0.65	0.63	0.48
hOrg_41d	0.19	0.94	0.93	0.95	1.00	0.93	0.92	0.94	0.65	0.64	0.48
hOrg_53d	0.19	0.94	0.94	0.94	0.93	1.00	0.95	0.96	0.67	0.66	0.55
hOrg_58d	0.21	0.94	0.94	0.94	0.92	0.95	1.00	0.96	0.69	0.68	0.58
hOrg_65d	0.20	0.94	0.94	0.95	0.94	0.96	0.96	1.00	0.68	0.67	0.55
Luo et al. 2016 CO_40d	0.22	0.65	0.66	0.65	0.65	0.67	0.69	0.68	1.00	0.98	0.86
CO_60d	0.22	0.63	0.64	0.63	0.64	0.66	0.68	0.67	0.98	1.00	0.88
Fetal_Ctx	0.20	0.48	0.49	0.48	0.48	0.55	0.58	0.55	0.86	0.88	1.00

Colour scale: 1.0, 0.5, 0, -0.5, -1.0

SUPPLEMENTARY FIGURE 2: Comparison of cerebral organoid proteomes with transcriptomics of the developing brain and cortical cerebral organoids. Spearman correlation of 45-day cerebral organoid proteome compared to **(A)** human fetal 8-37 post conceptional week, dorsolateral prefrontal cortex (DFC) and **(B)** human cortical organoids of 33 to 65 days *in vitro*.

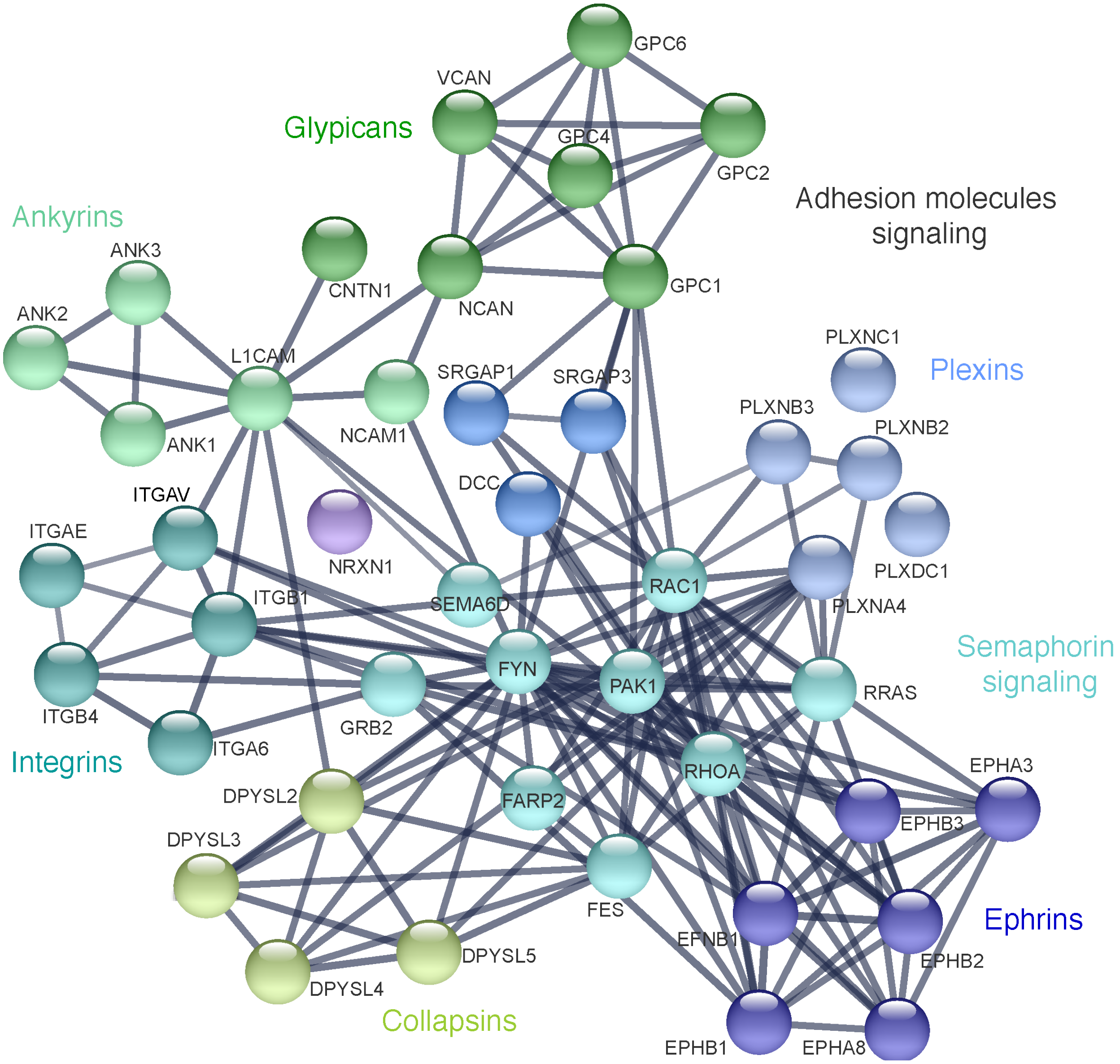

SUPPLEMENTARY FIGURE 3: Interactive organizational network of proteins from 45-day cerebral organoids. Molecular relationships between proteins found in cerebral organoids from the dataset are based on the STRING database (https://string-db.org/). Groups of proteins are separated by different colors, as indicated.
